# Large irritation fibroma of hard palate: a case report of a rare clinical entity

**DOI:** 10.11604/pamj.2021.38.61.27662

**Published:** 2021-01-19

**Authors:** Alexios Tsikopoulos, Charalampos Festas, Athanasios Fountarlis, Varvara Sidiropoulou, Nikolaos Chaitidis, Asterios Symeonidis, Angeliki Kotsiafti, Theano Papazi

**Affiliations:** 1 Department of Otorhinolaryngology, School of Medicine, University of Thessaly, Larissa, Greece,; 2 Department of Otorhinolaryngology, 401 Army General Training Hospital, Athens, Greece,; 3 Department of Histopathology, Theageneio Anticancer Hospital, Thessaloniki, Greece,; 4 Department of Internal Medicine, 401 Army General Training Hospital, Athens, Greece,; 5 Department of General Surgery, 424 Army General Training Hospital, Thessaloniki, Greece,; 6 Department of Hematology, University of Epirus, School of Medicine, University of Thessaly, Ioannina, Greece,; 7 Department of Otorhinolaryngology, Giannitsa General Hospital, Pella, Greece

**Keywords:** Benign tumor, irritation fibroma, hard palate, case report

## Abstract

Fibromas are benign tumors of connective tissue common in the oral cavity but rare on hard palate. This paper reports on an asymptomatic, slowly growing mass on the hard palate of a 90-year-old lady, with a reported use of denture for two decades. The patient presented with a 2.2cm, smooth-surfaced, well-circumscribed nodule attached with a stalk to the palatal mucosa. After excision, the histopathological examination revealed a mass of fibrous connective tissue, covered by stratified squamous epithelium with focal low-medium grade hyperplasia and hyperkeratosis. These findings were consistent with irritation fibroma of hard palate, a rare entity, which should be considered as a possible diagnosis for tumors of the area by every physician.

## Introduction

Fibromas are benign tumors of fibrous connective tissue [1]. Their size is usually small and their diameter is rarely larger than 1.5 centimeter [2]. Generally, fibromas are solitary, asymptomatic, sessile lesions, affecting patients between third and sixth decade of life [3]. They are found in 1.2% of adults and they have a 66% female predominance [4]. The association between fibromas and trauma is well established. Most of the fibromas of the oral cavity are reactive hyperplasia in response to local irritation or trauma [5]. They are common on gingiva, tongue and buccal mucosa [6]. However, although fibromas are common in the oral cavity, their incidence on the hard palate is rare, mainly because of fewer chances of trauma or irritation. This case report presents a patient with a fibroma located at hard palate of unexpectedly large size and aims to offer a brief overview of this rare clinical entity to every physician responsible for the diagnosis and treatment of tumors of the oral cavity.

## Patient and observation

A ninety-year-old lady presented to the Department of Otorhinolaryngology at Giannitsa General Hospital, Giannitsa, Greece with a chief complaint of a hard, large mass on the hard palate. History revealed that the patient first noticed the appearance of a small lesion (approximately 2cm in diameter) over 10 years ago and had been growing gradually yearly. This lesion was painless without causing any obstruction to mastication and speech. The patient stated however that in the last months there were problems with the application of a denture, causing constant irritation and pain in the region. The patient had no other specific symptoms. Her oral hygiene was remarkably poor. There was no history of infection or trauma around her anterior hard palate. Of note, the chronic use of the denture could have been implicated in the irritation of the palatal mucosa. The medical history of the patient was not a contributing factor (no alcohol consumption, passive smoker). The patient suffered from chronic obstructive pneumopathy, coronary disease and arterial hypertonia under medication.

**Figure 1:**
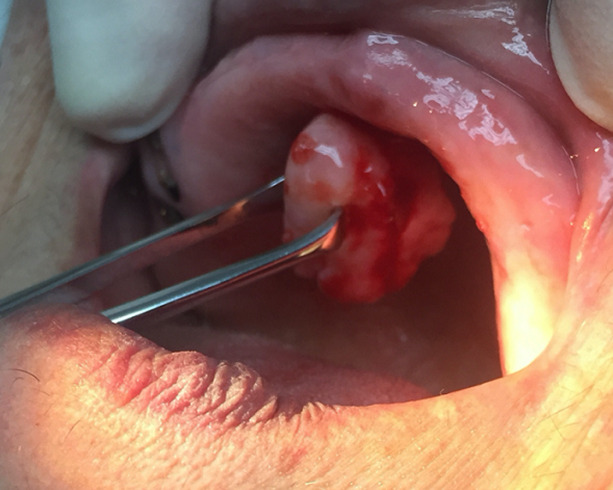
intraoral examination (before excision)

**Figure 2:**
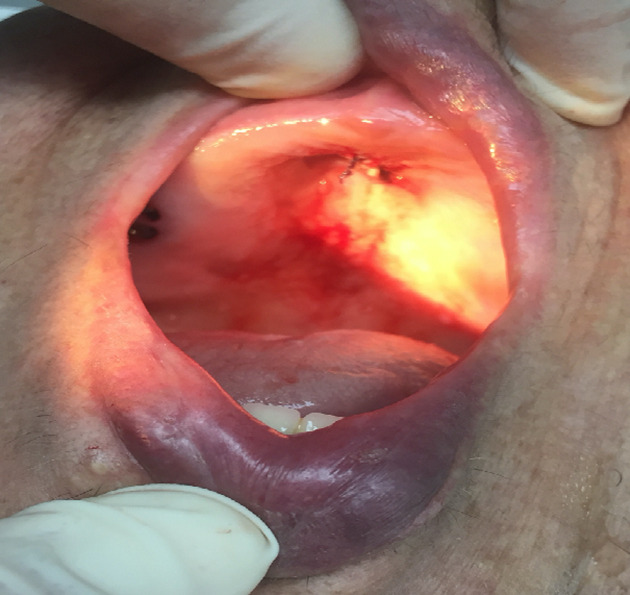
intraoral examination (after excision)

**Figure 3:**
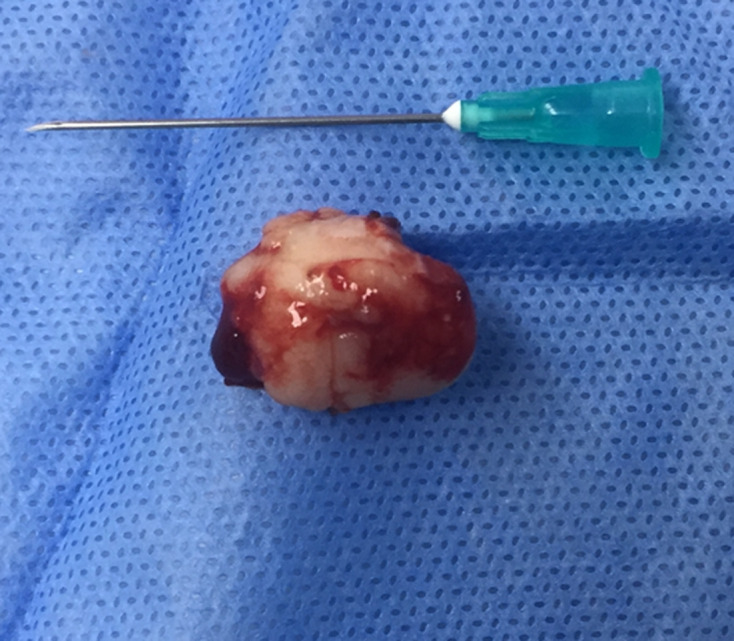
inferior aspect of the specimen

An intraoral clinical examination revealed a 2.2cm, smooth-surfaced, well-circumscribed nodule in hard palate (Figure 1, Figure 2, Figure 3). On palpation the tumor was hard and attached with a stalk to the palatal mucosa. Cervical lymph nodes were not enlarged and palpable. Preoperative routine blood investigation was within normal limits. No imaging studies were requested as it was deemed that they were not necessary. The tumor was to be differentiated from various palatal swellings on both clinical and histopathological grounds. Palatal fibroma, pyogenic granuloma, torus palatinus, dermoid cyst, ameloblastoma, squamous papilloma, oral lipoma and neurofibroma were considered under differential diagnosis. The lesion was totally excised under local anesthesia without complications and was sent for histological examination. No post-operative complications occurred. Because of the benign nature of the tumor, no further examinations were carried out.

**Figure 4:**
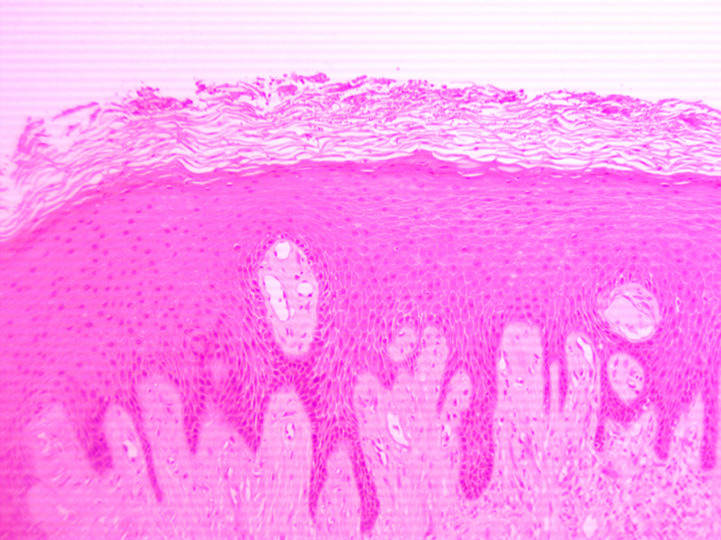
histopathological feature of the specimen (Zoom 10x), epithelium with hyperplasia and hyperkeratosis

**Figure 5:**
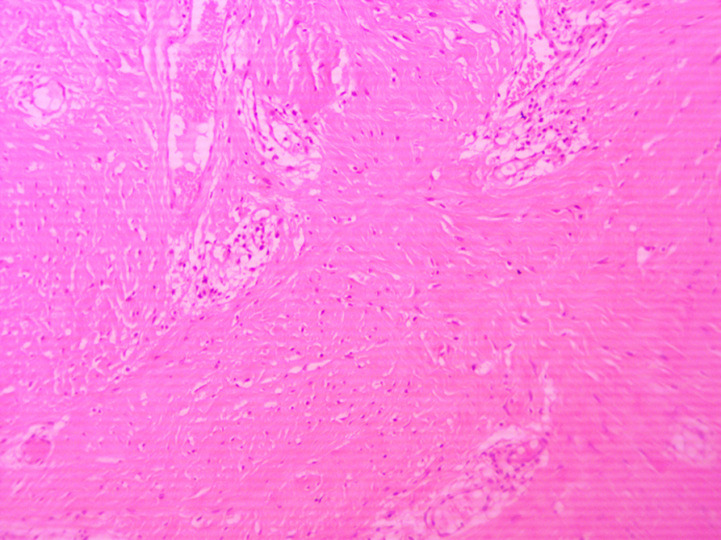
histopathological feature of the specimen (Zoom 10x), hyperplastic endothelium and perivascular inflammation

The histopathological examination revealed a nodular mass of fibrous connective tissue, covered by stratified squamous epithelium with focal low-medium grade hyperplasia and hyperkeratosis. Beneath the epithelium were spotted many small blood capillaries scattered all over the lesion and mild inflammation. These features were consistent with irritation fibroma (Figure 4, Figure 5). On the follow-up appointments, no signs of recurrence of the tumor were observed and the patient did not mention any further complaint.

## Discussion

Fibromatous lesions are benign tumors of extremely dense and collagenized fibrous connective tissue [7]. It is speculated that the etiology of fibroma is trauma to the affected mucosa. Thus, they tend to occur in sites predisposed to chronic, repeated irritation and trauma [5]. This was the case with the ninety-year-old patient of our study. Although there was no history of direct trauma, the continuous irritation of the palate mucosa because of the chronic use of denture led to the formation of this tumor on the hard palate.

As far as size is concerned, most fibromas are 1.5cm or less in diameter [8]. The present tumor was of a slightly larger diameter than expected. Continuous irritation and low-grade trauma lead to large fibroma because there is a lot of space to accommodate this asymptomatic tumor [5]. It was assumed that the small initial fibroma, under continuous irritation from the long-term daily application of a denture, slowly became larger over the course of a decade, since the patient had first noticed a small lesion about 10 years in hard palate.

Additionally, fibromas are mostly asymptomatic, they do not have any malignant potential and their recurrence is rare after a total excision [3]. In our case, the clinical features of an asymptomatic, benign, firm, solitary, well-circumscribed nodule, hard in palpation, were in comply with a fibroma and its diagnosis was easily established. Furthermore, there was no recurrence of the tumor found during the clinical examination of the patient on the programmed 6-month checkup.

Of great interest was the location of the fibroma. Fibromas can grow in all organs, are frequently found in the buccal mucosa, and are the most common tumor of the oral cavity [9]. However, hard palate is an unusual site of chronic irritation and trauma, and consequently of an irritation fibroma [6]. Furthermore, the histopathological analysis revealed a mass of fibrous connective tissue, covered by stratified squamous epithelium with focal low-medium grade hyperplasia and hyperkeratosis. These findings are typical for an irritational fibroma and were in comply with the histologic criteria of fibroma defined by Barker & Lucas [10,11]. According to them, irritation fibromas comprise a nodular mass of fibrous connective tissue covered with stratified squamous epithelium [12]. Overall, irritating fibromas of the hard palate are a rare entity and the current case report provides an overview of such a case to every clinical doctor responsible for the diagnosis or treatment of tumors of the oral cavity.

## Conclusion

Irritation fibroma of hard palate is a rare clinical entity. The current case report sought to provide information about a patient with an intraoral mass of the palatal mucosa of unusual size, so as to set irritation fibroma of hard palate among the possible differential diagnoses of similar tumors for every physician involved in the management of intraoral tumors.
